# In-Depth Characterization of Laser-Welded Aluminum-and-Copper Dissimilar Joint for Electric Vehicle Battery Connections

**DOI:** 10.3390/ma15217463

**Published:** 2022-10-25

**Authors:** Sajid Ali, Joonghan Shin

**Affiliations:** 1 Department of Future Convergence Engineering, Kongju National University, 1223-24 Cheonandaero, Seobuk-gu, Cheonan 31080, Korea; 2 Department of Mechanical and Automotive Engineering, Kongju National University, 1223-24 Cheonandaero, Seobuk-gu, Cheonan 31080, Korea

**Keywords:** laser welding, electric vehicle (EV), intermetallic compounds, dissimilar, aluminum, nickel-coated copper

## Abstract

With advancements in the automotive industry, the demand for electric vehicles (EVs) has remarkably increased in recent years. However, the EV battery, which is a vital part of the EV, poses certain challenges that limit the performance of the EVs. The joining of dissimilar materials for different components affects the electrical and mechanical performances of EV batteries. Laser beam welding is a promising technique for joining Al and Cu for application in secondary battery fabrication because of the precise control over heat input and high process speed. However, the production of Al–Cu joints remains challenging because of the differences between their thermal and metallurgical properties and the resulting formation of brittle and hard intermetallic compounds, which reduce mechanical and electric properties. Thus, it is vital to characterize the weld to improve joint performance and enhance the laser welding process. This study investigates the joining of an Al alloy (AA1050) with Ni-coated Cu using a continuous-wave Yb fiber laser. The evaluation of the weld morphology showed a correlation between the weld characteristics and process parameters (laser power and welding speed). The weld interface width and penetration depth into the lower sheet (Cu) both increased with increasing heat input. Optical microscopy of the weld cross-section revealed many defects, such as voids and cracks. Scanning electron microscopy (SEM) with energy-dispersive X-ray spectroscopy (EDS) was employed to examine the weld microstructure. The composition analysis revealed the presence of mixed morphology of Al–Cu eutectic alloy (α-Al+Θ-Al2Cu) phase in the form of dendrites in the weld fusion zone with traces of the highly brittle Al4Cu9 phase at a high heat input condition. Furthermore, the electrical contact resistance of the weld seam was measured to determine the correlation between heat input and resistance. In addition, Vickers microhardness measurements were performed on the weld cross-section to validate the SEM/EDS results.

## 1. Introduction

Recently, the automotive sector has witnessed a remarkable shift from conventional internal combustion engines (ICEs) to electric vehicles (EVs)/hybrid-electric vehicles [1,2]. EVs have almost net-zero emissions, making them a better alternative to traditional ICE-based vehicles [3,4]. Electric batteries, which are a vital part of EVs, currently have low efficiency and low energy density, which limits the driving range compared with ICE-based vehicles [5]. Hence, battery efficiency and energy density must be improved to improve the performance of EVs [6,7].

The EV battery pack consists of several thousand battery cells interconnected using tabs and busbars of dissimilar materials; typically aluminum and copper, in the case of Li-ion batteries. Thus, the reliable joining of different connections and the materials used is of enormous importance in the fabrication of EV battery packs, which directly affects the overall performance of the vehicle’s battery system. Li-ion rechargeable batteries are commonly used as power sources for EVs and many other electronic devices [8]. EV batteries are available in three different types based on the cell shape: cylindrical, prismatic, and pouch [9]. Usually, tab and busbar interconnectors in Li-ion batteries are manufactured using steel, aluminum, copper, and nickel-plated copper [10]. To obtain good fuel efficiency and reduce air pollution, lightweight metals and alloys are employed in various manufacturing applications [11]. Aluminum alloys and copper have been extensively used in various industries, including automotive, ship, and aerospace industries, owing to their high strength-to-weight ratio and good electrical, mechanical, thermal, and corrosion resistance [12,13].

Several joining techniques, such as laser welding, friction stir welding, tungsten inert gas welding, ultrasonic wire bonding, and resistance spot welding, have been employed to join aluminum and copper interconnectors in secondary battery manufacturing [14]. Among these techniques, laser beam welding is considered one of the most promising methods for joining aluminum and copper, owing to its easy automation, high precision, small heat-affected zone, contactless process, high process speed, and possibility of easy welding of dissimilar metals. Compared with other joining techniques, good electrical and mechanical performances have been reported using laser welding [15,16,17,18]. However, laser welding remains challenging in the joining of dissimilar materials, owing to its high reflectivity, low solubility, and differences in the properties of the joining materials. The low solubility leads to the formation of highly brittle intermetallic compounds (IMCs) after solidification of the weld seam, resulting in the embrittlement of the weld. Many studies have revealed that the formation of brittle IMCs must be minimized to obtain reliable joints between dissimilar metals [19]. Therefore, intermixing of the base metals must be minimized, which can be achieved by reducing the interaction time and controlling the penetration of the upper material into the lower one.

Many authors have investigated the laser welding of dissimilar materials; however, most have stressed the role of process parameters for producing a sound laser-welded joint. Vincenzo et al. [20] investigated the effect of spot diameter on the continuous-wave (CW) laser welding of 0.3 mm thick Cu electroplated with a thin Ni layer with 0.45 mm pure Al sheets. The authors found that a smaller spot diameter could produce sound weld beads with better control of penetration depth. Good electrical and mechanical properties were obtained by optimizing the process parameters. Trinh et al. [21] studied the characteristics of nano-second pulsed-wave (PW) laser welding of a 0.087 mm thin Al tab and 0.4 mm steel battery case for Li-ion secondary batteries. Several blowholes have been observed on the weld bead surface, while voids have been observed on the weld cross-section. The authors also explained the phenomenon of upward penetration owing to the recoil pressure in the melt pool. Shmalen et al. [22] evaluated a novel laser braze-welding of 0.2 mm Al and 0.5 mm Cu in a lap configuration with a 30 mm overlap. The authors obtained a wide process window for good laser process robustness and suggested a reduction in the intermixing of base metals to enhance joint strength. Solchenbach et al. [23] studied the laser braze-welding of 500 μm Al and Cu sheets using spatial power modulation of a fiber laser. The beam modulation was found to be effective in homogenizing the temperature distribution at the interface between Al and Cu. Further investigations showed that shear strength was directly linked to the thickness of the IMCs. The relationship between the mechanical characteristics and IMC layer thickness of Al–Cu laser-welded joints was also studied by Zuo et al. [24], who found that varying the heat input produced intermediate layers of different thicknesses at the interface, which controlled the shear strength of the joint. It was revealed that the presence of eutectic and hypoeutectic phases resulted in weak joints. Yan et al. [25] studied CW/PW dual-beam laser welding of steel/aluminum sheets. The authors suggested the applicability of dual-beam welding for the reduction of blowholes in welded joints. Microstructure analysis revealed the formation of a 10 µm IMC layer at the interface. Weigl et al. [26] studied the effect of filler material in the Cu–Al laser welding and found that the filler material promoted more uniform intermixing, which significantly enhanced the ductility of the joints. Similarly, Chen et al. [27] studied Cu–Al welding using Ni–P-coated Al to obtain Al–Cu joints without brittle IMCs. The obtained welds were donut-shaped and free from detrimental Al–Cu IMCs; weld defects were also inhibited. Huang et al. [28] investigated the metal mixing phenomenon in the laser keyhole welding of dissimilar metals using experimental and numerical modeling approaches. They found that recoil pressure contributes to the upward flow of the melt pool, resulting in the migration of Cu from the bottom to the top. Marangoni waves generate a backward flow and two side vortices that promote the intermixing of the base metals. Furthermore, it was reported that laser power is directly related to both recoil pressure and Marangoni stress, while increasing the welding speed inhibited the mixing of base metals in the fusion zone.

The above literature survey reveals that many studies have been conducted on laser-welded joints of dissimilar metals. However, further studies are needed to evaluate the effects of laser process parameters on quality of joints in terms of weld morphology and weld defects. In this study, laser lap welding of Al (AA1050) and thin Ni-coated Cu was conducted. The effects of laser power and welding speed on the weld morphology and weld defects were investigated using optical microscopy. Subsequently, the microstructure and composition of the weld zone were characterized using scanning electron microscopy (SEM) with energy-dispersive X-ray spectroscopy (EDS), and the electrical properties of the joint were evaluated by measuring the electrical contact resistance of the weld joint. Additionally, the mechanical properties of the weld were determined using the Vickers microhardness test.

## 2. Materials and Methods

In this experimental study, 70 mm × 40 mm plates of Cu (>99.5%, 1.5 mm thick) coated with a thin layer of Ni (3 μm) and Al alloy (AA1050, 0.75 mm thick) were welded in a lap configuration. Table 1 lists the chemical composition of the AA1050 alloy.

**Figure 1:**
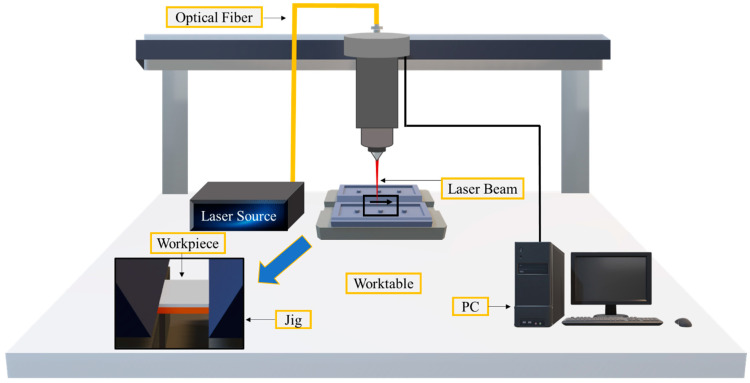
Schematic of experimental setup.

**Figure 2:**
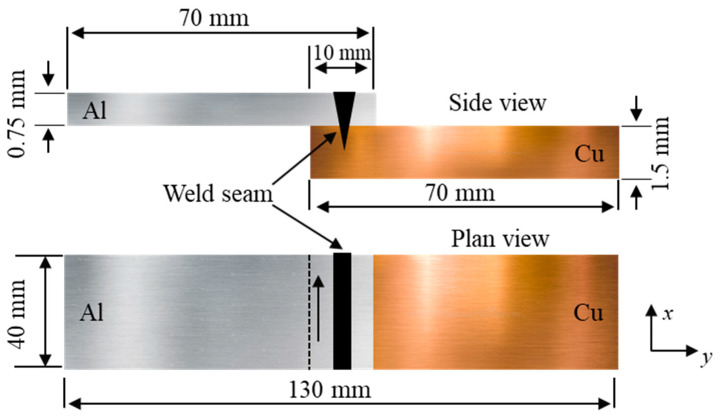
Schematic of the lap welding configuration.

Figure 1 illustrates the experimental setup and clamping device (jig). The samples were arranged in a lap-joint configuration by positioning aluminum on top of copper with a 10 mm wide overlap, as shown in Figure 2. The samples were cleaned with ethanol to remove impurities from the surfaces of the plates. A single-mode continuous-wave Yb: fiber laser (Raycus RFL-C2000) with a maximum power output of 2000 W and wavelength of 1080 nm was employed for welding. The laser beam was delivered by an optical fiber and focused on the specimen’s surface using a focusing lens with a focal length of 182 mm. The beam size focused on the workpiece surface was 100 μm. In laser lap welding, full penetration of the laser beam into the top sheet is required to achieve complete fusion of the base metals. For this, a preliminary bead-on-plate test was conducted by gradually varying the speed and laser power to determine the process conditions necessary for achieving full penetration of the laser beam onto the Al plate.

To obtain the full penetration conditions for the Al plate, three experimental groups named A, B, and C were designed with laser powers of 1500, 1600, and 1700 W, respectively, and the scan speed was varied from 15 to 30 mm/s in 5 mm/s increments for each power level. Laser welding was performed with the beam focused on the top surface of the Al plate using the experimental process parameters listed in Table 2.

After laser welding, the samples were preliminarily observed using optical microscopy (AM4515 Series, Dino Lite) to examine the weld surface. To characterize the weld microstructure and weld geometry, specimens (15 mm × 10 mm) were prepared by diamond-cutting the welded samples at the center point. Standard metallographic samples were prepared by cold mounting the specimens in resin and subsequently grinding them using SiC papers, followed by polishing in diamond suspensions of 3 and 1 µm.

**Figure 3:**
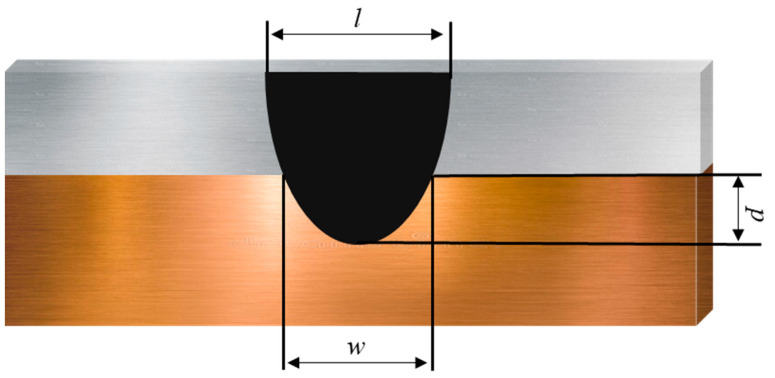
Characteristics of weld geometry.

Optical microscopy was performed to investigate the cross-sectional morphology of laser-welded joints. The weld geometry of the cross-section (see Figure 3) was characterized by measuring the top bead width *(l*), penetration depth (*p*), and weld interface width (*w*). Microstructure analysis of the weld cross-sections was performed using SEM (TESCAN VEGA3) equipped with EDS. The weld seam microstructure was examined using SEM, and EDS was employed to perform elemental composition analysis of the IMCs in the weld fusion zone (FZ).

**Figure 4:**
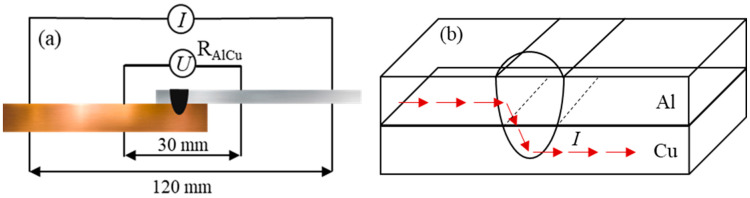
Electrical contact resistance measurement: (**a**) schematic; (**b**) current flow direction.

The electrical contact resistance of the weld joint was measured using the four-wire method [15]. The measurements were performed using a Keithley Integra Series Model 2460 ohmmeter with a measurement current of 100 mA. Each measurement was performed by applying a current for 60 s and then taking the average value of the measured resistance. Measurements were performed at a testing length of 30 mm across the weld joint. A schematic of the resistance measurement setup is shown in Figure 4a, and the current flow direction is illustrated in Figure 4b.

**Figure 5:**
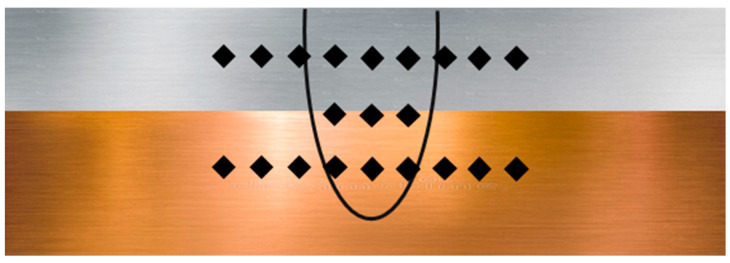
Vickers microhardness indentation positions.

Microhardness was measured using a Vickers microhardness tester (Mitutoyo HM-100 series) to investigate the mechanical properties of the weld. Measurements were performed at a 50-gf load and 10 s dwell time. Indentations were carried out horizontally along the weld cross-section at three different positions, as shown in Figure 5.

## 3. Results and Discussion

### 3.1. Analysis of the Weld Morphology and Defects

**Figure 6:**
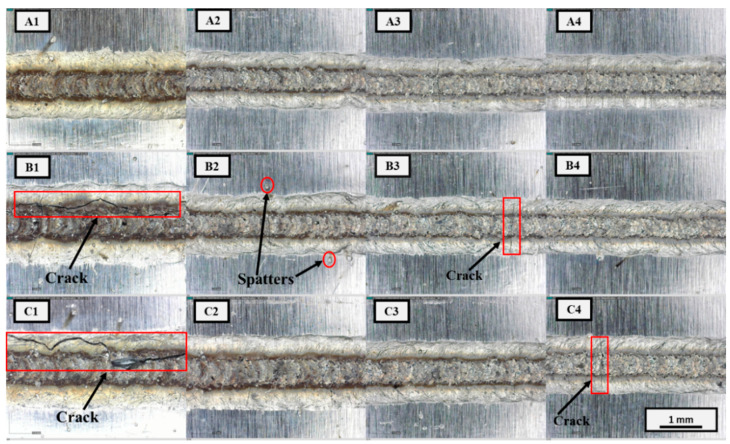
Optical micrographs of top weld bead: (**A1**) power = 1500 W, welding speed = 15 mm/s; (**A2**) power = 1500 W, welding speed = 20 mm/s; (**A3**) power = 1500 W, welding speed = 25 mm/s; (**A4**) power = 1500 W, welding speed = 30 mm/s; (**B1**) power = 1600 W, welding speed = 15 mm/s; (**B2**) power = 1600 W, welding speed = 20 mm/s; (**B3**) power = 1600 W, welding speed = 25 mm/s; (**B4**) power = 1600 W, welding speed = 30 mm/s; (**C1**) power = 1700 W, welding speed = 15 mm/s; (**C2**) power = 1700 W, welding speed = 20 mm/s; (**C3**) power = 1700 W, welding speed = 25 mm/s; (**C4**) power = 1700 W, welding speed = 30 mm/s.

Optical micrographs of the top beads of the weld joints are shown in Figure 6. Under varying parameters, laser welding yielded weld joints of uniform bead width. The bead width tended to increase with laser power and decrease with increasing welding speed. The correlation between the bead width, laser power, and welding speed can be explained by the change in the heat input because of the changes in parameters. Optical microscopy of the weld joint revealed the presence of surface defects, such as spatter, while cracks were formed at a relatively high heat input as a result of hot cracking inside the melt pool. Surface cracks were clearly visible in groups B and C, while in group A no cracks were observed; however, spatters were observed around the weld joint. Overall, the severity of cracking tended to decrease with decreasing heat input. As well as the heat input, cracks can also be affected by power density (applied laser energy per unit area and time). Group C including C4 used the higher power density condition (see Table 2) compared with other groups (A and B). This high power density condition may be the reason for the crack generation in C4, though the heat input is relatively low.

**Figure 7:**
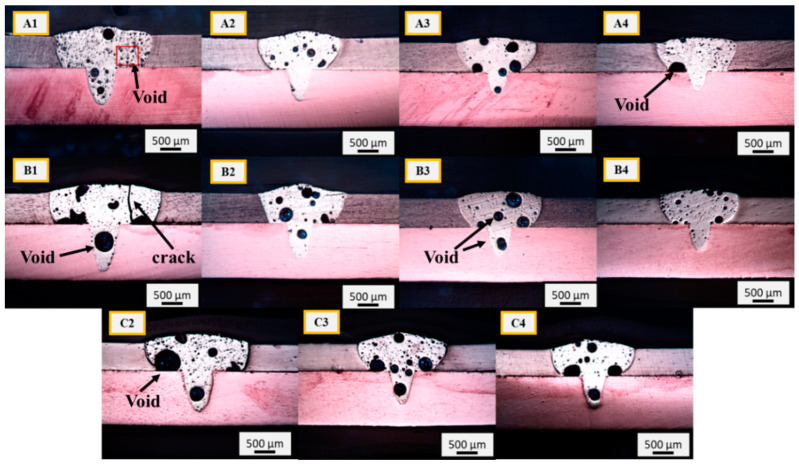
Optical micrographs of the weld transverse cross-section; (**A1**) power = 1500 W, welding speed = 15 mm/s; (**A2**) power = 1500 W, welding speed = 20 mm/s; (**A3**) power = 1500 W, welding speed = 25 mm/s; (**A4**) power = 1500 W, welding speed = 30 mm/s; (**B1**) power = 1600 W, welding speed = 15 mm/s; (**B2**) power = 1600 W, welding speed = 20 mm/s; (**B3**) power = 1600 W, welding speed = 25 mm/s; (**B4**) power = 1600 W, welding speed = 30 mm/s; (**C2**) power = 1700 W, welding speed = 20 mm/s; (**C3**) power = 1700 W, welding speed = 25 mm/s; (**C4**) power = 1700 W, welding speed = 30 mm/s.

Cross-sectional images of the welds obtained using optical microscopy are shown in Figure 7. All samples show a fully developed weld in the Cu sheet. The laser beam irradiation melted Al, and the molten weld metal penetrated the Cu sheet through the keyhole mode. The top part of the weld FZ exhibits a convex profile with a high width-to-depth ratio, whereas the lower part of the FZ exhibits a low width-to-depth ratio. This is attributable to the low melting temperature of Al, which resulted in a wide FZ in the top part, owing to the high energy density of the laser beam. Further observations revealed a large number of weld defects, such as cracks and voids of different sizes. At a high heat input, the temperature inside the melt pool increased above the vaporization point of Al, which generated a recoil pressure [21]. The recoil pressure inside the melt pool tends to render the melt pool flow more turbulent. This pressure and atmospheric gases trapped inside the FZ during welding lead to the formation of voids after solidification. The formation of porosity/void in the laser welding is often caused by metallurgical factors, such as dissolved hydrogen in the melt pool. Other than the trapping of gas, environmental factors also influence porosity formation, such as air temperature, air humidity, and dissolved oxygen. Increasing both air temperature and humidity results in increased porosity [29]. In addition to other factors, heat input in laser welding also affects porosity formation. As can be seen in Figure 7, compared to high heat input conditions, the porosity level in samples such as A2, A4, and B4 is low. It is pertinent to mention here that heat input is the dominant factor for a high degree of porosity in this type of welding. Because of the high heat input, more energy is accumulated in the weld metal, resulting in an unstable and turbulent melt pool flow. The recoil pressure generated in the melt pool coupled with the turbulent melt pool flow leads to porosity in the weld metal due to the trapping of dissolved gases that form bubbles in the melt pool. Therefore, the high porosity in group C and some of the group B samples can be attributed to the high heat input. In the case of large heat input, longitudinal cracks are observed, as can be seen in sample B1, owing to the possible formation of brittle IMCs and the thermal cycling effect that made the weld prone to hot cracking. Crack formation can also be caused by the difference in the coefficients of thermal expansion of the welded materials.

**Figure 8:**
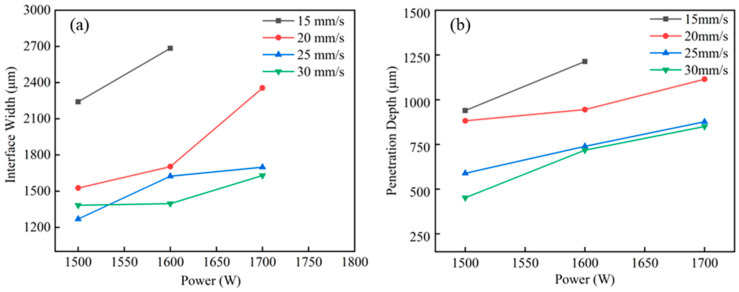
Interface width and penetration depth as a function of laser power: (**a**) interface width; (**b**) penetration depth.

The weld joint was characterized by measuring the weld geometry. The penetration depth (*p*) into the lower Cu sheet was measured from the interface between the two materials, whereas the weld interface width (*w*) was measured at the interface between the two sheets, as depicted in Figure 3. The measured penetration depth and weld interface width were plotted against the power (Figure 8). With increasing power, both the penetration depth and the weld interface width tended to increase. According to Figure 8a, at 20 mm/s, the weld width increases from 1525.57 µm at 1500 W to 2355.46 µm at 1700 W, whereas, at other speeds, a slight change in the weld width with an increase in power is observed. In the case of 15 mm/s scan speed, a relatively large increase in the interface width was found. The scan speed of 15 mm/s resulted in the highest heat input, as shown in Table 2. The rate of increase in the heat input was 33.3% when the scan speed changed from 20 mm/s to 15 mm/s. However, when the scan speed changed from 25 mm/s to 20 mm/s and from 30 mm/s to 25 mm/s, the rates of increase in the heat input were only 25% and 20%, respectively. It is considered that the relatively large increase in the heat input induced the large increase in the interface width. As shown in Figure 8b, the penetration depth correlates with the laser power and welding speed. As is evident from Figure 8, the penetration depth tends to increase with laser power and decrease with increasing welding speed. The conditions used in this study yielded a weld seam with partial penetration, which eventually reduced the intermixing of the base metals and the corresponding formation of IMCs, thus improving the joint strength. As reported in [20,27], the partial penetration of the weld in the lower base metal resulted in less mixing of Cu and the weld metal, which reduced the formation of brittle and hard IMCs. It can be concluded that low heat input is favorable for minimizing the generation of detrimental IMCs, which eventually enhances joint strength.

### 3.2. Analysis of the Weld Microstructure and Composition

**Figure 9:**
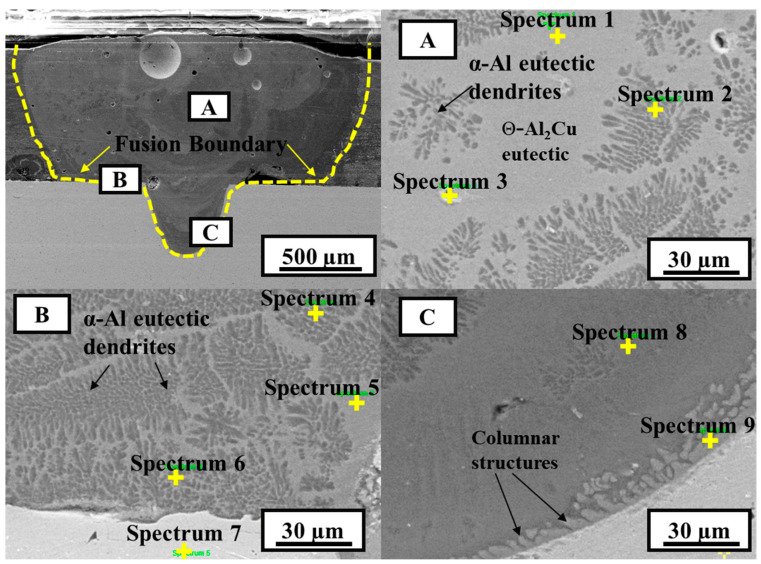
SEM images of sample A4 (power = 1500 W, welding speed = 30 mm/s); (**A**,**B**,**C**) are the magnified SEM images of microstructures at location A, B and C in weld fusion zone, respectively.

**Figure 10:**
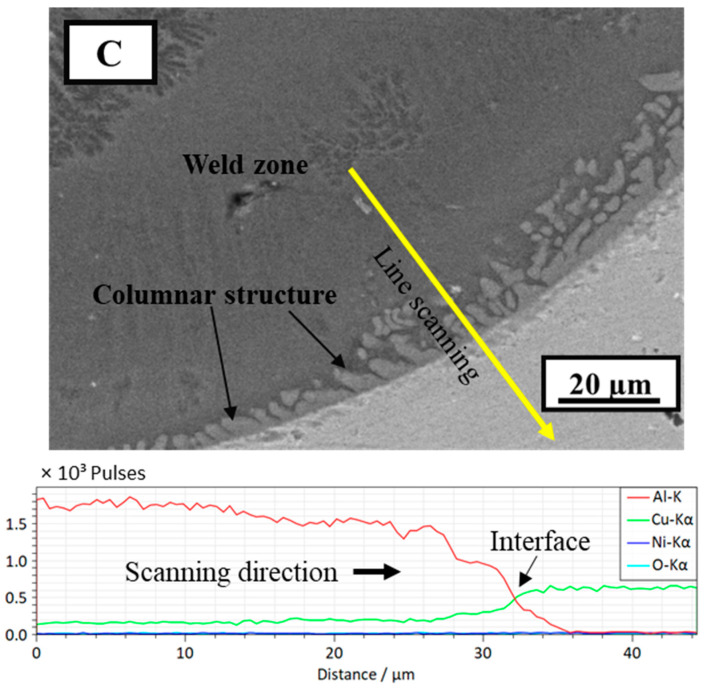
SEM/EDS line scan of region C in Figure 9 (power = 1500 W, welding speed = 30 mm/s).; (C) is the magnified SEM image of location C in weld fusion zone in Figure 9.

The microstructure of the selected low- and high-heat-input welds was analyzed. Figure 9 shows the SEM images of sample A4 corresponding to the low heat input condition, and the results of the EDS point and line scanning analysis are shown in Table 3 and Figure 10, respectively. Different zones in the weld FZ are indicated by letters A, B, and C, as shown in Figure 9. According to the Al–Cu binary phase diagram, the composition analysis given in Table 3 shows that the weld metal is entirely an Al–Cu eutectic alloy (α-Al+Θ-Al_2_Cu) with 6.38–20.18 at.% Cu. In the upper part of the FZ, as indicated by letter A, a mixed morphology of dark dendrite (α-Al eutectic) and gray structure (Θ-Al_2_Cu eutectic) phases is observed (see Figure 9). The EDS point analysis results show a high content of Al in the FZ, which is an indication of the Al–Cu eutectic phase. Near the Al–Cu interface, indicated by letter B, the microstructure is a mixture of gray and dark structures (a dark dendritic structure is more dominant than the gray structure). Region B is close to the Al–Cu interface, and the presence of a darker dendrite structure (α-Al eutectic) is likely due to the Ni layer that inhibited the diffusion of Cu into the weld metal in this region. Point analysis of spectra 4, 5, and 6 shows similar chemical compositions, suggesting a similar type of phase formation as that of region A. The composition corresponding to spectrum 7 indicates a solid solution of Cu with 89.85 at.% Cu with traces of Al and Ni. Near the FZ–Cu interface, indicated by letter C, the composition is the same as those of A and B; however, morphological changes in the shape of columnar structures are observed near the interface. It is expected that, at the FZ–Cu interface, the diffusion of Cu into the weld metal intermixing led to the growth of the columnar morphology. The evolution of different microstructures is controlled by different parameters, including cooling rate and alloy composition of the weld pool. In the weld pool, these parameters significantly vary from region to region and process to process, which makes it quite challenging to predict the microstructures of the weld zone. However, columnar grains usually grow in a direction that is opposite to the cooling direction, and they can grow further when the cooling rate is relatively low.

**Figure 11:**
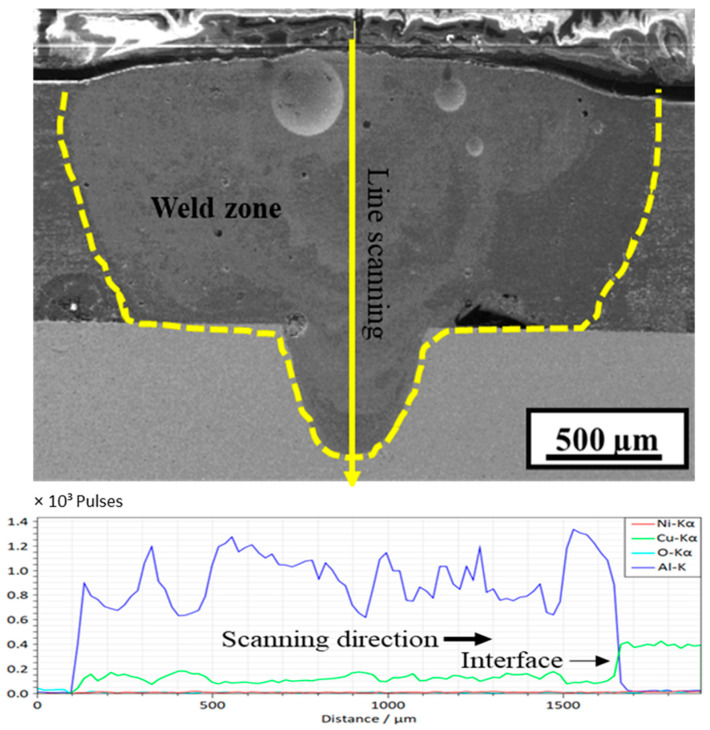
SEM/EDS vertical line scan of sample A4 (power = 1500 W, welding speed = 30 mm/s).

To further analyze the IMC formation, EDS line scanning was performed at the FZ–Cu interface, and the results are shown in Figure 10. The line scanning indicates an almost uniform distribution of Al and Cu in the FZ; however, the concentration gradient at approximately 24–32 µm along the scanning path shows a decrease in Al and an increase in Cu content, which hints at the IMC phase change. A similar type of line scanning was also performed vertically along the FZ from top to bottom, as shown in Figure 11. Compared to the line scanning at the interface, the vertical line scanning results show non-uniform distributions of Al and Cu, with the Al content dropping to almost zero at the interface. In summary, the analysis results provide an overall distribution of constituent elements in the weld and the probability of IMC formation inside the weld and at the interface.

**Figure 12:**
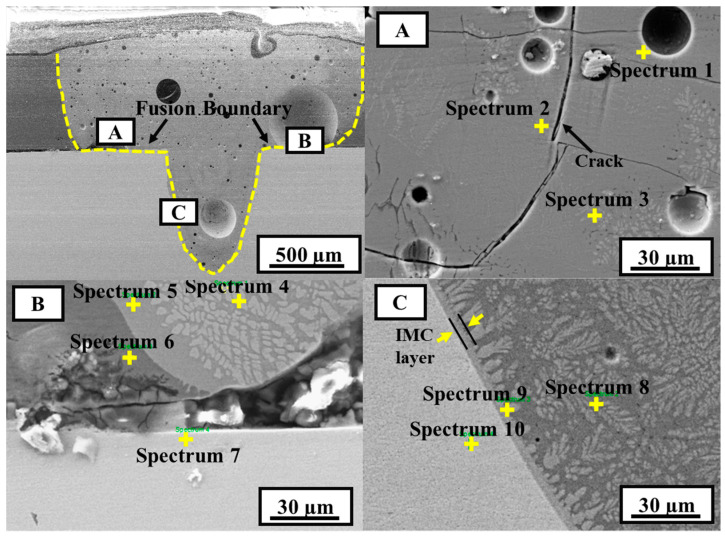
SEM images of sample C2 (power = 1700 W, welding speed = 20 mm/s); (**A**,**B**,**C**) are the magnified SEM images of microstructures at location A, B and C in weld fusion zone, respectively.

SEM images of sample C2 (high heat input) are shown in Figure 12, and the chemical compositions of the marked points in different zones are listed in Table 4. It is evident from the appearance of the weld that the penetration depth of Al is greater than in sample A4. The number and size of the defects are also notably higher in sample C2. A magnified view of region A in Figure 12 clearly shows voids or pores of varying sizes. Weld cracks are also observed in the same area. These cracks can possibly be hot cracks, which mostly formed through the generation of unbearable shrinkage stress during the solidification of the molten metal. This type of crack is very common during the fusion welding of nonferrous materials. However, this type of cracking can also occur because of the formation of highly brittle IMCs after the solidification of the melt pool. Because dissimilar welding of Al and Cu involves the generation of brittle IMC phases, crack generation is likely caused by these IMCs. It can be observed that the average Cu content in the weld metal is slightly higher than under low heat input. The composition of the marked spectrum 2 in region A shows an increased Cu content (76.84 at.% Cu), which clearly confirms the formation of a brittle Al_4_Cu_9_ phase near the crack. The results of spectra 1 and 3 suggest the possible presence of an Al–Cu eutectic alloy (α-Al+ Θ-Al_2_Cu), similar to previous results. Region B, near the Al–Cu interface, also shows the type of morphological appearance and composition very similar to the previous sample. The enlarged SEM image of region B also exhibits defects in this region. Using the Al–Ni binary phase diagram, the presence of Ni_3_Al can be detected in spectrum 7, owing to the Ni interlayer. In region C, at the FZ–Cu interface, a thin continuous layer of IMC is observed in the form of columnar dendrites, as indicated in Figure 12. The results of spectrum 8 show a similar composition to the previous cases, whereas spectrum 9 shows the possible presence of the Θ-Al_2_Cu eutectic phase. At the same time, at the position of spectrum 10, the composition is 100% Cu.

**Figure 13:**
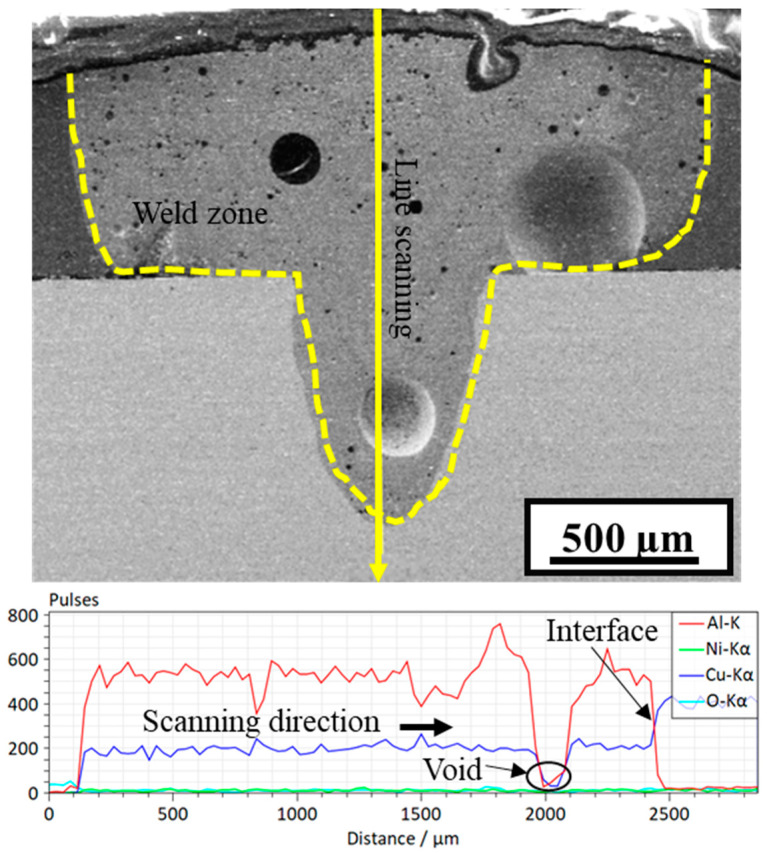
SEM/EDS vertical line scan of sample C2 (power = 1700 W, welding speed = 20 mm/s).

Figure 13 shows the EDS line scan of the weld FZ along the vertical direction from top to bottom. The Cu content (blue line) here is slightly higher than in sample A4, indicating the enhanced diffusion of Cu in the FZ. The line-scanning results reveal a non-uniform distribution of Al and Cu along the scanning path. At approximately 2000 µm of the line scan, both the Al and Cu contents sharply drop. This can be attributed to the formation of a large void, which can also be observed in the image. At the interface, the Al content drops to zero, while that of Cu significantly increases.

Based on the SEM and EDS results, sample C2 took the highest heat input, while sample A4 took the lowest heat input. With the increase in heat input by altering the welding parameters, the diffusion kinetics increased, enhancing the amount of Cu inside the weld zone. Furthermore, the penetration depth of the melted Al also increased with heat input. Although the high heat input increased the penetration depth of the weld metal into Cu, it also introduced a higher amount of weld defects, such as porosity and weld cracks, leading to the formation of brittle IMCs. When joining dissimilar metals, the formation of IMCs is inevitable to form a metallurgical bond. The IMC amount and phases directly affect the mechanical and electrical properties of the joint; hence, the formation of IMCs must be controlled by minimizing interdiffusion. A high heat input increases the weld melt pool temperature, further enhancing diffusion, resulting in a higher amount of detrimental IMCs, solute segregation, and brittleness in the weld metal. Thus, it is advisable to perform laser welding at a low heat input.

### 3.3. Vicker’s Microhardness Test

**Figure 14:**
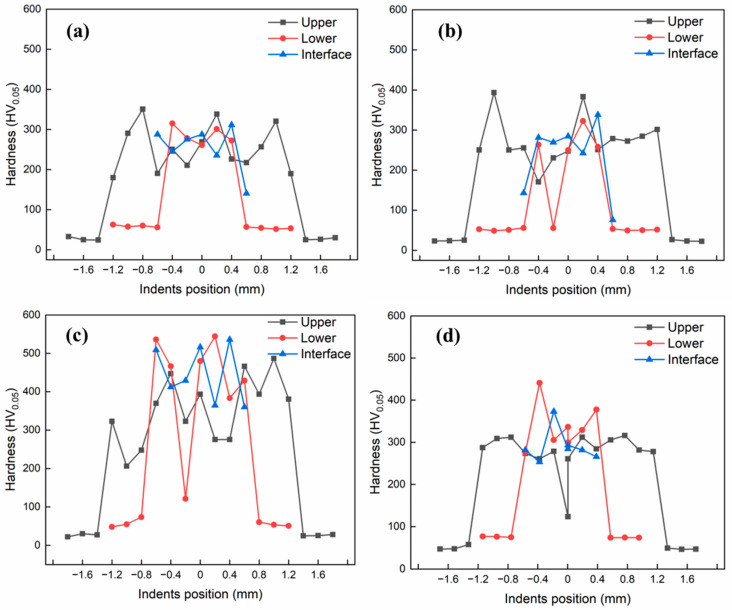
Hardness profile as a function of the distance from the weld center: (**a**) A4; (**b**) B4; (**c**) C2; (**d**) C4.

To characterize the mechanical properties of the weld joint, microhardness was measured using the Vickers hardness method. Figure 14 shows the Vickers microhardness test profiles as a function of distance from the weld center. To prevent deformation by adjacent indentations, the indent spacing was at least three times larger than the measured diagonal length of the first indentation. In Figure 14a, the microhardness profile corresponding to specimen A4 (low-heat input condition) is presented. Evidently, the weld FZ has higher hardness than the base metals, which can be attributed to the local microstructure and formation of brittle and hard IMC phases. The increased hardness can also be explained by the enhanced ionic and covalent bonds, and reduced metallic bonding in the weld metal zone, as reported in [23]. The microhardness values of the Al and Cu base metals were 24 and 54 HV, respectively, whereas the hardness of the weld was in the range of 140–310 HV. This hardness value corresponds to the less brittle Al_2_Cu IMC phase (Table 5), which is vital for the metallurgical bonding of the base metals in dissimilar welding. Local variations in hardness inside the FZ are observed owing to the presence of pores and voids.

Figure 14b shows the microhardness profile corresponding to sample B4. As can be seen from the figure, the hardness profile is almost the same as that of sample A4. However, a slight fluctuation in the local hardness distribution can be observed possibly due to the evolution of different microstructures and porosity formation in the weld metal. Figure 14c depicts the microhardness profile of sample C2 (high heat input condition), where a significant increase in hardness value (up to 530 HV) can be observed. This increase in microhardness is in line with the results obtained from the SEM/EDS analysis. The high heat input led to the increased mixing of Cu into the FZ; thus, possibly leading to the formation of the Cu-rich Al_4_Cu_9_ phase (see Table 4), based on the EDS composition results in the preceding section (see Table 4). The presence of the brittle phase caused the formation and propagation of cracks in the FZ, as can be observed in the SEM image of the sample (see Figure 12). Similar to the previous sample, the microhardness of the FZ is higher than that of the base metals. Furthermore, the hardness profile shows that microhardness values at the interface and lower part of the FZ are higher than that in the upper part of the FZ. Figure 14d presents the hardness profile corresponding to sample C4. The maximum hardness value observed was 441 HV, while the local minimum hardness value was 105.1 HV in the upper region, possibly due to the presence of voids. In summary, the microhardness in the FZ is higher than those of the base metals because of the formation of hard and brittle IMC phases and microstructural evolution. More brittle and hard phases were detected at high heat inputs because of the increased diffusion of Cu inside the FZ and subsequent intermixing in the weld metal.

### 3.4. Electrical Contact Resistance

**Figure 15:**
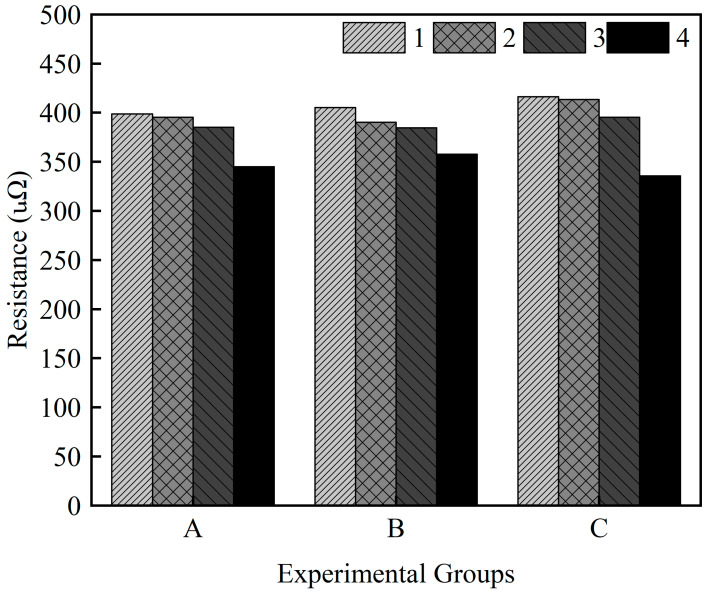
Electrical contact resistance.

The results of the electrical contact resistance measurements of the weld joint are shown in Figure 15. The resistance was measured following the four-point method: the principle of the resistance measurement is explained in [15]. The resistance correlated with the sample number and legends 1, 2, 3, and 4 show an increase in the welding speed from 15 to 30 mm/s for each experimental group, as shown in Figure 15. The measured resistance is in the range of 335.60–416.28 μΩ and the lowest resistance is observed for sample C4 (laser power of 1700 W and welding speed of 30 mm/s). Samples C1 and C2 exhibit the highest values, which is likely due to weld defects and weld morphology. No significant differences in the measured resistance between the groups have been detected; however, the resistance tended to decrease with increasing welding speed within the same group. The high resistance in the case of high heat input is also characterized by greater mixing of Cu in the weld, resulting in the formation of more brittle IMCs with high resistivity. With increasing penetration depth into Cu, the amount of Cu mixed in the weld is expected to increase, and thus, more Cu-rich brittle IMCs can be formed in the FZ [30]. It is vital to note that electrical contact resistance has a great impact on the overall performance of the battery. In summary, the formation of IMCs significantly enhances the electric resistance owing to the increased mixing of the base metals. Moreover, weld defects and weld morphology also affect the electrical contact resistance of the joint.

## 4. Conclusions

In this study, thin Al sheets (AA1050) were welded with thick Cu sheets using CW fiber laser welding with varying laser power and welding speed. The morphology, microstructure, electrical contact resistance, and mechanical properties of the welds were evaluated. The experimental results are summarized as follows:

- The laser power and welding speed affected the weld characteristics. Weld defects, such as voids and cracks, appeared on the weld cross-sections.
- SEM/EDS of the weld microstructure revealed that the weld metal consisted of mixed-morphology dark (α-Al eutectic) and gray (Θ-Al_2_Cu eutectic) phases.
- At a high heat input, a more brittle Al_4_Cu_9_ IMC phase was formed, which likely caused the formation of cracks in the weld metal.
- The electrical contact resistance tended to decrease with increasing welding speed; however, the difference in resistance was negligible with varying laser power.
- The Vickers microhardness test validated the IMC phase identification in the weld metal. Moreover, the hardness of the weld metal was higher than that of the base metals, indicating the possibility of IMC formation.

## Figures and Tables

**Table 1:** AA1050 chemical composition (wt.%).

Si	Fe	Cu	Mn	Mg	Zn	Ti	V	Other	Al
0.070	0.300	0.010	0.020	0.010	0.010	0.020	0.020	0.019	Bal.

**Table 2:** Laser process conditions used in the experiment.

Group	Sample No.	Laser Power (W)	Scan Speed (mm/s)	Heat Input (J/mm)	Power Density (10^6^ W/cm^2^)
A	A1	1500	15	100.0	19.11
	A2		20	75.0	19.11
	A3		25	60.0	19.11
	A4		30	50.0	19.11
B	B1	1600	15	106.7	20.38
	B2		20	80.0	20.38
	B3		25	64.0	20.38
	B4		30	53.3	20.38
C	C1	1700	15	113.3	21.66
	C2		20	85.0	21.66
	C3		25	68.0	21.66
	C4		30	56.7	21.66

**Table 3:** Chemical composition of zones denoted in Figure 9.

Spectrum	Al (at.%)	Cu (at.%)	Ni (at.%)	Possible Phase
1	91.84	8.16	0	Al–Cu eutectic
2	79.82	20.18	0	Θ-Al_2_Cu
3	93.44	6.56	0	Al–Cu eutectic
4	92.45	6.71	0.84	Al–Cu eutectic
5	93	7	0	Al–Cu eutectic
6	91.20	8.80	0	Al–Cu eutectic
7	7.75	89.85	2.4	Cu (Solid solution)
8	92.16	6.82	0.74	Al–Cu eutectic
9	92.11	6.38	1.51	Al–Cu eutectic

**Table 4:** Chemical composition of zones denoted in Figure 12.

Spectrum	Al (at.%)	Cu (at.%)	Ni (at.%)	Possible Phase
1	83.86	16.14	0	Al–Cu eutectic
2	23.16	76.84	0	Al_4_Cu_9_
3	85.63	14.37	0	Al–Cu eutectic
4	86.78	13.22	0	Al–Cu eutectic
5	88.68	11.32	0	Al–Cu eutectic
6	95.26	4.74	0	Al–Cu eutectic
7	25.83	0	74.17	Ni_3_Al
8	84.74	15.26	0	Al–Cu eutectic
9	77.16	22.84	0	Θ-Al_2_Cu
10	0	100	0	Cu (Solid Solution)

**Table 5:** Characteristics of intermetallic compounds [20].

Phase	Nominal Composition	Chemical Composition [%at. Cu]	Electrical Resistivity [µΩ cm]	Hardness [HV]
(Al)		0–2.2	2.4	36
Θ	Al_2_Cu	31.9–33	8.0	630
ƞ	AlCu	51.9–54.8	11.4	905
ζ	Al_3_Cu_4_	56–57.5	12.2	930
ƴ	Al_4_Cu_9_	64–69	14.2	770
(Cu)		80.3–100	2.0	75

## Data Availability

The data presented in this study are available upon request from the corresponding author.
